# Menopause knowledge and attitude among Iranian women

**Published:** 2015

**Authors:** M Taherpour, F Sefidi, S Afsharinia, JH Hamissi

**Affiliations:** * Midwifery Department, Qazvin University of Medical Science, Qazvin, Iran; ** Department of Psychometrics, Qazvin University of Medical Sciences, Qazvin, Iran; *** Dentist; **** Department of Periodontics and Preventive Dentistry, College of Dentistry, Qazvin University of Medical Sciences, Qazvin, Iran

**Keywords:** knowledge, promotion, menopause

## Abstract

**Objective:** The present study was done to assess the effects of training on knowledge and attitude level promotion of post menopause women about menopause.

**Methods & Material:** The research communication included 100 menopausal women aged 45-60 years, who were selected in a stratified manner (according to the economic status: poor, average, and good). The tool used was an examiners-made questionnaire, which contained 3 parts: demographic characteristics, knowledge and attitude measurement.

**Results:** Eleven percent of the studied women had a low knowledge regarding menopause and 1% was good. After training, 27% got a good knowledge and no one remained at the low level. The attitude of 59% of the studied women regarding menopause was positive and 80% got a positive attitude after training. A significant relation was found between knowledge and attitude, before and after training.

**Conclusion:** Despite the fact that the majority of women judge menopause as a positive incident, it seems that paying attention to their training caused the bringing to their knowledge of the natural menopause and having a healthy and jolly life.

## Introduction

Menopause is a combination of two Greek words, meno (monthly), and pause (stop), it means the stopping of the menstruation and the ending of fertility. The natural age of menopause is 45-55 years and its main cause is the stopping of the natural activity of the ovaries. It would also occur following the ovaries extraction by surgical operation [**1**]. Menopause, the cessation of menstruation, is a psychosocial and biological event. The psychosocial phenomena surrounding menopause are attitudes, perceptions and expectations. Clinically, menopause represents the cessation of the monthly cycles. It is a transitional period in women’s life [**2**]. Due to the decrease in the production of estrogen, there are some symptoms of menopause such as vaginal dryness, intercourse problem, joint pain, muscle pain, back pain, skin thinning, and dryness, fornication, osteoporosis (bone problems), sleep problems (insomnia) or disturbed sleep, urinary problems, sensations, forgetfulness, hot flushes and other vasomotor symptoms. The psychological symptoms or effects include anxieties, mood swings, and emotional problems [**2**]. Research conducted by Enrighe (2003), Adodo (2004) and Senwna (2008) revealed that the daily intake of 800-1500 mg of calcium, the consumption of the dairy products such as milk, cheese and yogurt, fish liver oil, farm fresh products such as wheat germ, grain and vegetable, should be taken into account. The consumption and drinking of plenty of water and a decrease in smoking, caffeine consumption, and alcoholic intake can prevent osteoporosis and increase bone strength during menopause. Hormone replacement therapy (HRT) can be used to replace the loss and decline in estrogen hormone production [**3**,**4**]. Attitude refers to feelings, beliefs, and reactions of an individual towards an event, phenomena, object, or person. Attitudes are not innate attributes of mankind, they are learnt responses (Adewuyi, 2006) [**5**].

The most common symptoms reported by women during the menopause transition are hot flushes and night sweats, which affect approximately 70% of women in Europe and North America. However, the prevalence of vasomotor symptoms and the experience of menopause vary considerably between cultures and countries [**6**,**7**]. Cultural differences have been explained by differences in attitudes and meanings of menopause, such as the extent to which menopause is seen as a medical condition or a natural phenomenon, or whether mid-life represents positive or negative social changes and/ or values within a society [**8**].

Additional explanations offered by researchers for the differences in symptom experience within and between cultures include diet, body mass index, exercise and mood, as well as attitudes towards menopause [**9**-**12**]. Despite the interest in the role of attitudes, few studies have explicitly examined the relationships between attitude and symptom experiences [**13**,**14**]. Perception, attitudes and knowledge regarding the menopause and its transitional period, the climacteric, may differ from one female population to another. These differences have been related to female age, parity and hormonal status as well as social, economical, cultural, educational, and geographical factors [**15**-**23**].

Pan believed that knowledge is a basic requirement for the use of health services and attitude is an organizing principle for doing an action and it can trigger a health behavior due to its affection. Creation of knowledge and positive attitude is effective and reasonable to continue changes in behavior, albeit it should overcome the main obstacles [**24**]. Although some women had wide information about the physiological changes of menopause in Bertro’s study (2003), their knowledge about self-care, prevention, or reduction of these symptoms and problems was low. He believed nurses and midwives could have the greatest role in training the middle-aged women during menopause to treat or reduce its symptoms and signs [**25**]. Regarding the importance of health during menopause and the negative attitude of Iranian women toward the menopause phenomenon, we decided to assess the effect of training on knowledge and attitude of post menopause women regarding menopause in Qazvin, in 2009.

## Methods & Material

This intervention study research communication implied postmenopausal women aged 45-60 years, living in Qazvin, Iran. Blocks were selected stratified (according to the economic states: poor, average, and good). According to the previous study, in order to determine the sample size we considered p=0.5, accuracy=0.1 and assurance=95% and sample size=121. After training, 100 people completed questionnaires and were enrolled. The samples were selected randomly. If elected people were not eligible to enter the study, another sample was chosen. Inclusion criteria were age of 45-60 years, at least one year after the last menstrual period, not having history of chronic disease surgical operation or psychiatric disorder. The exclusion criteria included: received previous training regarding menopause, artificial menopause, severe stress in past 6 months, the risk of chronic disease, having a knowledgeable person regarding the problems of menopause in the family.

After selecting the eligible samples, the researchers went home and provided the necessary explanation regarding the purpose of the study. The informed consent was obtained from all the samples and their demographic information was recorded, then information about the knowledge and attitude of samples was collected by using the questionnaire. The questionnaire included three parts of demographic information and questions about knowledge and attitude. The part of knowledge measurement contained 23 questions and knowledge of sample was divided into 3 categories: weak (score<8), Middle (8<score<16) and good (score>16).

The part of attitude measurement contained 14 multiple choice (completely agree to completely disagree) questions. Likert scale was used for scoring (5 = completely agree, 1 = completely disagree). Therefore, samples were divided into 3 categories: negative attitude (score 14-36) neutral (score 37-47) and positive attitude (score 48-80) (completely disagree or disagree people were in the negative attitude group and completely agree or agree people were in the positive attitude group). To determine the validity of date collection method we used content validity and its reliability was determined by a repeated test. This way, 10 women completed the questionnaire in a 10 days interval. The correlation coefficient was 94%.

Before starting the training, a primary test was taken and obtained information was assessed, then the training needed was determined and an appropriate curriculum was developed. The training content included nature, complications, and symptoms of menopause, prevention, and treatment of problems. At the end of this program, the questions of the studied sample were answered. After 2 months, the secondary test was taken.

**Statistical Analysis **

Data were collected by using the paired t test and ANOVA at a significant level of 05/ 0 P ≤ statistical analyses were used.

## Results

In this study, the mean age of the samples was 47.97 ± 3.47 years. 84%of them were jobless and 16% were occupied. The education level of the majority was diploma (32%) and just 5% had a higher education. Family income level of many samples was average (64%). 85% of the studied persons did not use hormone replacement therapy **Table 1**.There was a significant difference in the knowledge and attitude regarding menopause, before and after training (p less than 0.001).

**Table 1:** Characteristics of the study sample (N=100)

Variable	Level	%
Age	>49	71
	50-54	23
	<55	6
Educational level	Illiterate	16
	Primary	23
	Intermediate	24
	Diploma	32
	University	5
Employment	Yes	16
	No	84
Income	Weak	21
	Moderate	64
	Good	15
HRT	Yes	15
	No	85

 As it can be seen in **Table 2**, there was a significant increase in the average of the knowledge score from 10.52 to 15.14.**Table 3** indicates that 88% of the samples had moderate knowledge about menopause and just 1% had good knowledge. However, after training, 27% got good knowledge. Result showed that the rate of poor awareness of people was decreased from 11% to Zero after training.

**Table 2:** Comparison between knowledge and attitude of samples before and after training

Variables	after training	before training	Significant
	mean± SD	mean± SD	
Knowledge	15.14 ± 2	10.52 ± 2.43	p<0.001
Attitude	49.92 ± 7	47.49 ± 7.37	p<0.001

**Table 3:** Distribution of knowledge and attitude of individuals regarding menopause, before and after training

Distribution of knowledge and attitude		Knowledge				Attitude			
	Intervention	weak	moderate	good	sum	negative	Neutral	positive	Sum
Before training	number	11	88	1	100	7	34	59	100
	percent	11	88	1	100	7	34	59	100
After training	number	0	73	27	100	3	17	80	100
	percent	0	73	27	100	3	17	80	100

This finding also indicated that attitudes regarding menopause in 59% of the samples was positive before training and negative in 7%, but it got to 10% in positive attitude and just 3% had a negative attitude after training. There was not any significant relation between the knowledge of samples and their demographic variables such as age, education level, and occupational income. Moreover, there was not any relation between attitude and age, education level and income before and after training.

## Discussion

The finding of this study showed that knowledge of 11% studied women regarding menopause was poor and just 1% had a good knowledge. After training, 27% got good knowledge and no one remained weak. The attitude of 59% of the studied samples regarding menopause was positive and it increased to 80% after training. All the researchers who studied menopause would emphasize on training and caring of this group to prevent their problems [**26**,**27**]. The result of the study of Patricia et al. in Ecuador showed that 79.4% of the women were concerned regarding menopause and followed health care in this field and, 77.9% of them had an attitude of changing life style and receiving health care services. 49% believed that they should obtain a proper knowledge and 93.1% wanted more information regarding menopause [**28**]. In the study in Italy (Serena et al, 2009), 90% of the studied persons accepted menopause as a natural period in the women’s life and more than 40% expressed it as a good experience. More than half of the samples did not have any information regarding menopause and its possible treatments [**29**]. The study of Bertro and Tsao et al. showed that knowledge about different aspects of menopause is poor [**25**,**30**]. In the study by Adewuyi and Akinade in Nigeria, 64.5% of the studied sample had good knowledge [**2**]. In a study done in America, the knowledge of most people (80% ) regarding menopause was good and 48.5% of then sought for more information regarding the menopause phenomenon [**31**]. In other studies, the knowledge of more than half of the samples were good [**32**,**33**]. The knowledge level of the studied people was average and it seemed that the differences existed because of cultural and socioeconomic differences. In a survey of Mazhar (2003), in Pakistan, 74.3% of the participants, expressed they needed some training regarding menopause and the prevention of postmenopausal problems [**34**]. In a study by Rolinck et al. in south America, which assessed the effect of training on the knowledge of middle-aged women on osteoporosis (the most important complication of menopause), the average score for knowledge was 3 +/- 1.53 before training but it got to an average of 99.9 +/- 2.65 after training, albeit the education level of 80% of the participants in this study was diploma or higher. In the present study, although there was a little increase in the average score of knowledge, it was just due to the low level of education status of subjects. After taking training courses on complications of osteoporosis, its prevention and treatment, most people changed their behavior regarding diet, exercise and calcium intake. 43% increased their Vitamin D intake and 14% had started their drug therapy. According to the researchers, training on post menopause could encourage women change their health behaviors [**35**].

Hence, the task of health workers, the training of health care, was manifested. In this study, the relationship between education and knowledge before and training was not significant, which was in contrast with other studies [**32**,**36**]. The reason would be the low number of highly educated samples; the majority of the subjects having an education level under diploma.

Ten studies were reported on women’s attitudes; according to 6 of these studies, women who described menopause as a natural life transition had positive attitudes [**37**-**42**] but 4 studies did not mention whether attitudes were positive or negative [**43**-**46**]. Having no idea showed the lack of knowledge regarding menopause. Further researches in other countries reported positive attitude of the subjects.

In one survey, the attitude of most women regarding menopause and the cessation of menstruation was sense of obviation; they thought became an experienced and positive person [**16**]. Koster (1991) also reported that 66% of the Dornish women’s attitude toward menopause was the feeling of obviation; in this study, the closer to menopause the more positive attitude existed in women [**33**]. In a study by Adewuyi and Akinade in Nigeria, 61% of the women had a positive attitude regarding menopause [**2**]. Findings also indicated that after learning about the attitude it was sufficient to great knowledge, attitudes being formed. In this study, there was not any significant relation between education and attitudes before and after training, which was in contrast with other studies [**16**]. This might be the reason for the lack of a significant relationship between knowledge and education discussed here.

Most authors, who discussed the theory of training a patient (Client), encouraged health care workers to follow the principles of teaching and learning. Evaluation is one of the most important steps of the training program for patients [**47**]. Today, client training has changed from training to education, conceptually. Especially when the measurement of learning comes out, behavior changes and corporation of clients in making decisions about the training process is very important. The corporation of a client in the process of training-learning is a fundamental philosophy in empowering clients [**48**].

## Conclusions

Health education has a significant impact on increasing knowledge, attitude, and practice of menopausal women to word postmenopausal complication. Hence, the improvement of health behavior in postmenopausal women during menopause should be emphasized on learning regarding the problems of menopause.

Goals of medicine, health promotion, health maintenance, and minimizing the suffering of people included in the prevention concept, and the most important step in prevention is health training. Since education is a tool for public health, according to the results obtained from this study and similar surveys, it was acknowledged that all women who experienced menopause should be trained. Improving knowledge regarding the natural menopause and changing attitude could change the behavior and improve their performance.

**Conflict of interest and funding**

The authors have not received any funding or benefits from industry in order to conduct this study.

**Acknowledgements**

The author would like to thank those menopausal women whose eagerness and willing cooperation to this survey, made it possible.
